# Comparison of standard mismatch repair deficiency and microsatellite instability tests in a large cancer series

**DOI:** 10.1186/s12967-024-04960-y

**Published:** 2024-02-13

**Authors:** Maja L. Nádorvári, István Kenessey, András Kiss, Tamás Barbai, Janina Kulka, Erzsébet Rásó, József Tímár

**Affiliations:** Department of Pathology, Forensic and Insurance Medicine, Semmelweis University, Budapest, Hungary

**Keywords:** Mismatch repair deficiency, Immunohistochemistry, Microsatellite instability, Pentaplex PCR, Malignant tumors

## Abstract

**Background:**

The tumor-agnostic indication of immune checkpoint inhibitors to treat cancers with mismatch repair deficiency (dMMR)/microsatellite instability (MSI) increased the demand for such tests beyond Lynch syndrome. International guideline recommendations accept immunohistochemistry (IHC) for dMMR or molecular techniques (PCR or NGS) for MSI status determinations considering the two tests are equal, although there are scattered reports contradicting to this presumption.

**Materials and methods:**

Here we have directly compared four protein MMR immunohistochemistry (IHC) to MSI Pentaplex PCR test in a large cancer patient cohort (n = 1306) of our diagnostic center where the two tests have been run parallel in 703 cases.

**Results:**

In this study we have found a high discrepancy rate (19.3%) of the two tests which was independent of the tumor types. The MSI PCR sensitivity for MMR IHC status was found to be very low resulting in a relatively low positive and negative predicting values. As a consequence, the correlation of the two tests was low (kappa < 0.7). During analysis of the possible contributing factors of this poor performance, we have excluded low tumor percentage of the samples, but identified dMMR phenotypes (classic versus non-classic or unusual) as possible contributors.

**Conclusion:**

Although our cohort did not include samples with identified technical errors, our data strongly support previous reports that unidentified preanalytical factors might have the major influence on the poor performance of the MSI PCR and MMR IHC. Furthermore, the case is open whether the two test types are equally powerful predictive markers of immunotherapies.

**Supplementary Information:**

The online version contains supplementary material available at 10.1186/s12967-024-04960-y.

## Background

Mismatch repair deficiency (dMMR) and its consequence, the microsatellite instability (MSI), [1, 2] may occur in any cancer type but it is typical in colorectal-, gastric- and endometrial cancers leading to a high tumor mutation burden (TMB) [3–5] and better antitumoral immune response [6, 7]. Since MMR gene defects can be inherited leading to Lynch syndrome, the diagnostics of this type of genetic defect was developed for that purpose: testing MMR proteins by IHC, followed by confirmatory testing the functional consequence, microsatellite instability, ultimately sequencing the MMR gene(s) [8, 9].

Development of the immune checkpoint inhibitor (ICI) therapies of cancer involved the development of predictive biomarkers like PDL1 (TPS and CPS) and associated companion diagnostics [10]. Since one of the major determinant of high TMB is the MSI status of the tumor, studies have been designed to evaluate the efficacy of ICI in MSI-high tumors [11] and found that these immunotherapies are barely active in microsatellite stabile (MSS) tumors leading to tumor-agnostic indications of several ICIs for MSI-high tumors [12–15]. However, the clinical trials and the drug registries used dMMR and MSI-high (MSI-H) as equally effective positive predictors for immunotherapy efficacy and international recommendations treat the two type of diagnostics equal [16–18] although dMMR is based on the detection of missing MMR protein while MSI test is based on the detection of the functional consequence of it at genome level. One of the major comparative analysis of dMMR IHC (four proteins) and MSI-PCR (Pentaplex panel) was performed on several thousands of colorectal cancer patients demonstrated a very low discrepancy rate (3.8%) [19, 20]. A recent meta-analysis of the incidence of MMR defects in colorectal- and gastric cancers documented a ~ 30% difference between dMMR and MSI-high determinations [21] suggesting that the two methods may not be fully equivalent. Furthermore, recent reports challenged the either/or testing protocol, mostly because of the unusual patterns of the dMMR as determined by immunohistochemistry [22, 23]. On the other hand, MMR IHC was standardized, the evaluation criteria are well defined by CAP and NICE (17, www.nice.org.uk/guidance/dg42) and MSI-PCR was improved from „Bethesda-panel” to a more sensitive mononucleotide Pentaplex panel [24–26] so a direct comparison of the two is feasible. Here we have used a large patient dataset where MMR protein and MSI tests have been run parallel allowing a direct comparison of the two methods.

## Materials and methods

Sample collection. Throughout 2018–2023 we have tested 1306 tumor samples for dMMR/MSI status where the two methods have been run parallel in 703 cases for the request of the interdisciplinary panel (Table 1). Our Department serves as molecular pathology center for primary and secondary oncology clinics, accordingly the waste majority of these cases were primarily diagnosed in local pathology departments. In 64 cases technical failures, mostly due to identified preanalytical factors (typically poor fixation) prevented the performance of the tests. The retrospective analysis of this cohort was approved by the local Ethic Committee of the Semmelweis University (RKEB62/2023).

**Table 1:** Characterization of the cancer patient cohort

	Male	Age mean ± SD	Female	Age mean ± SD	MMR and/or MSI tests	MMR and MSI tests
Cancers	701	65 ± 8.9	605	64 ± 9.2	1306	**703**
Colorectal	556	65 ± 8.8	422	65 ± 9.0	978	**543**
Endometrial			39	59 ± 9.3	39	**27**
Pancreatic	15	67 ± 7.4	21	61 ± 8.0	36	**14**
Gastric	22	67 ± 9.3	11	71 ± 9.2	33	**27**
Biliary	8	65 + 6.8	12	67 ± 6.8	20	**10**
Esophageal	13	65 + 6.5	3	54 ± 6.9	16	**13**
Ovarian			14	58 ± 10.2	14	**10**
Breast			11	51 ± 7.5	11	**4**
Liver	4	48 ± 7.1	6	71 ± 11.9	10	**3**
Others	17	60 ± 11.0	6	63 ± 12.1	23	**12**
CUP	66	63 ± 9.4	60	61 ± 9.4	126	**40**

Other cancers: appendiceal, nasopharyngeal, peritoneal, pharyngeal, prostate, small intestinal, testicular, thyroideal

*CUP* cancer of unknown primary, *MMR* mismatch repair protein, *MSI* microsatellite instability

MMR protein staining. Immunohistochemistry of the MMR proteins was performed on FFPE blocks by using ready-to-use mouse monoclonal antibodies of Ventana (Tucson, AR), anti- MLH1(M1), anti-MSH2 (G219-1129), anti-PMS2 (A16-4) and rabbit monoclonal anti-MSH6 (SP93). The immunoreaction was developed by the DAB Ultraview kit in the BenchmarkUltra automatic stainer (Ventana). Two types of positive controls have been used: in case of each antibodies a parallel positive control sample was used as well as in the tumor stroma fibroblasts and lymphoid cells served as inner controls. dMMR was reported as classical two-protein negativities (MLH1/PMS2 or MSH2/MSH6 losses), non-classical single- or multiple negativities in > 90% of tumor cells or as unusual (focal/subclonal or heterogenous negativities of > 10 to < 90% of tumor cells) in the background of positive stromal cells. In case of weaker tumor cell staining compared to normal cells or negative stromal cell nuclear staining, the case reported to be equivocal [27–29].

MSI-PCR. The specimen was processed to review its morphology on H&E stained slides and tumour and non-tumour areas were dissected separately from unstained sections for DNA extraction (High Pure PCR Template Preparation Kit–Roche). DNA was then amplified by PCR for five mononucleotide microsatellite markers (BAT-25, BAT-26, MONO-27, NR-21 and NR-24) and two pentanucleotide markers (Penta C and Penta D as internal technical controls for PCR) using Promega MSI Analysis System Version 1.2, followed by fragment analysis of the PCR products using ABI 3730 Genetic Analyzer. Genotype patterns of the MSS K562 human cell line and tumour samples were compared for each marker. Samples are defined as MSI-high if two or more of the five markers are unstable, MSI-indeterminate (MSI-low) if one marker is unstable and MS-stable if there is no detectable alterations of the 5 markers. The sensitivity of the test is 10% according to the manufacturer.

Statistics. During the comparison of MMR-immunohistochemistry to the MSI-PCR results, specificity, sensitivity, positive- and negative predictive values have been calculated. Furthermore, Kappa correlation coefficients were also calculated according to Cohen’s method with the cut-off value > 0.7 [30].

## Results

The patient cohort where in a significant proportion of the cases (703/1306) MMR-IHC and MSI-PCR tests have been run parallel is shown on Table 1. The main purpose of the tests was immunoncology indication. It is of note that MMR/MSI testings were most frequently run in colorectal cancer but interestingly the second most frequent cancer type was metastases of CUP tumors.

The incidence of MSI-H molecular status in our cancer cohort was 12.1% and was very similar in the colorectal part of the cohort. Interestingly, the dMMR incidence was proved to be higher, 20.3% in the entire cohort, and was very similar in the colorectal part. On the other hand, the incidence of pMMR and MSS were very similar in the entire cohort and in the colorectal part as well (Table 2).

**Table 2:** Incidence of various MMR/MSI categories in our cancer cohort

Cancer	MMR-IHC (n)	dMMR n (%)	pMMR n (%)	MSI-PCR (n)	MSI-H n (%)	MSI-L n (%)	MSS n (%)
All	1021	207 (20.3)	814 (79.7)	988	120 (12.1)	80 (8.1)	788 (79.8)
Colorectal	809	157 (19.4)	652 (80.6)	712	91 (12.8)	58 (8.1)	563 (79.1)
Non-colorectal	212	50 (23.6)	162 (76.4)	276	29 (10.5)	22 (8.0)	225 (81.5)

*dMMR* mismatch repair deficiency, *pMMR* mismatch repair proficiency, *MSI-H* microsatellite instability-high, *MSI-L* microsatellite instability-low, *MSS* microsatellite stability

Next, we have determined the discrepancy rate between MMR IHC and MSI PCR and found 19.3% discrepancy in the entire cohort. However, in case of pMMR the discrepancy rate for MSS/MSI-low was very low (2.0%) in the entire cohort and in the colorectal part as well. However, we have found that the discrepancy rate of dMMR versus MSI-high was very high in the entire cohort (60.9%) as well as in the colorectal cancer part (58.6%) and was even much higher in the non-colorectal cancers (Table 3).

**Table 3:** Comparison of MMR immunohistochemistry and MSI PCR results in our cancer cohort

	n	MSS n(%)	MSI-L n(%)	MSS/MSI-L n(%)	MSI-H n(%)	discrepancy n(%)
All cancers	703					136 (19.3)
pMMR	496	448 (90.3)	38 (7.7)	486 (98.0)	10 (2.0)	10 (2.0)
dMMR	207	108 (52.2)	18 (8.7)	126 (60.9)	81 (39.1)	126 (60.9)
Colorectal	543					98 (18.0)
pMMR	386	350 (90.7)	30 (7.8)	380 (98.4)	6 (1.7)	6 (1.6)
dMMR	157	79 (50.3)	13 (8.3)	92 (58.6)	65 (41.4)	92 (58.6)
Non-colorectal	160					38 (23.8)
pMMR	110	98 (89.1)	8 (7.3)	106 (96.4)	4 (3.6)	4 (3.6)
dMMR	50	29 (58.0)	5 (10.0)	34 (68.0)	16 (32.0)	34 (68.0)

*IHC* immunohistochemistry, *MMRD* mismatch repair deficiency, *MSI-H* microsatellite instability-high, *MSI-L* microsatellite instability-low, *MSS* microsatellite stability

We have compared the MSI PCR predictive value to MMR IHC. Statistical analysis indicated that the PCR has high specificity but low sensitivity, a moderate PPV but a lower NPV in case of colorectal or non-colorectal cancers (Table 4). The correlation tests indicated a modestly low rate between the two methods (kappa: ~ 0.5–0.3), not reaching the expected cut-off 0.7 (Table 4). When we have compared the predictive value of MMR IHC to MSI PCR test, we have found a very high NPV value but a very poor PPV one (Additional file 1: Table S1).

**Table 4:** Predictive power of MSI PCR for MMR IHC status

Cancer type	Sensitivity (%)	Specificity (%)	PPV (%)	NPV (%)	% Agreement	Kappa value
Colorectal	41.4	98.5	91.6	80.5	81.9	0.476
Non-colorectal	32.0	96.4	80.0	75.1	76.3	0.339

*kappa* Cohen’s kappa (95% CI), *NPV* negative predictive power, *PPV* positive predictive power

The performance of the MSI-PCR might be affected by the low tumor/normal (T/N) ratio of the analyzed tissue, where 20% is serving as diagnostic cut-off. Accordingly, we have excluded those cases from the analysis where the T/N ratio was lower than 20% and performed the comparative analysis. The discrepancy rate was found to be similar in this part of the cohort (18.9%) as well as in colorectal- (17.4%) and non-colorectal parts (24.8%) suggesting that the high discrepancy rate is not due to the tumors with low T/N ratio, since we have used macrodissection for compensation (Additional file 1: Table S2).

The performance of Pentaplex PCR is influenced also by the performance of the component markers. The analysis of the MSI-H tumors indicated that in case of colorectal cancer the five markers performed very similarly (84–97%) while in case of non-colorectal cancers two markers (N21, N24) exhibited a much lower levels (67–73%) as compared to the other three (Additional file 1: Table S3). A similar evaluation of the MSI-L cases indicated that in both colorectal and non-colorectal cancers BAT25 alteration is predominant (62.5 and 37.5%, respectively) followed by a lower frequency of N21 (21.9 and 25.0%, respectively), while the other three markers are much rarely altered (Additional file 1: Table S3).

**Fig.1:**
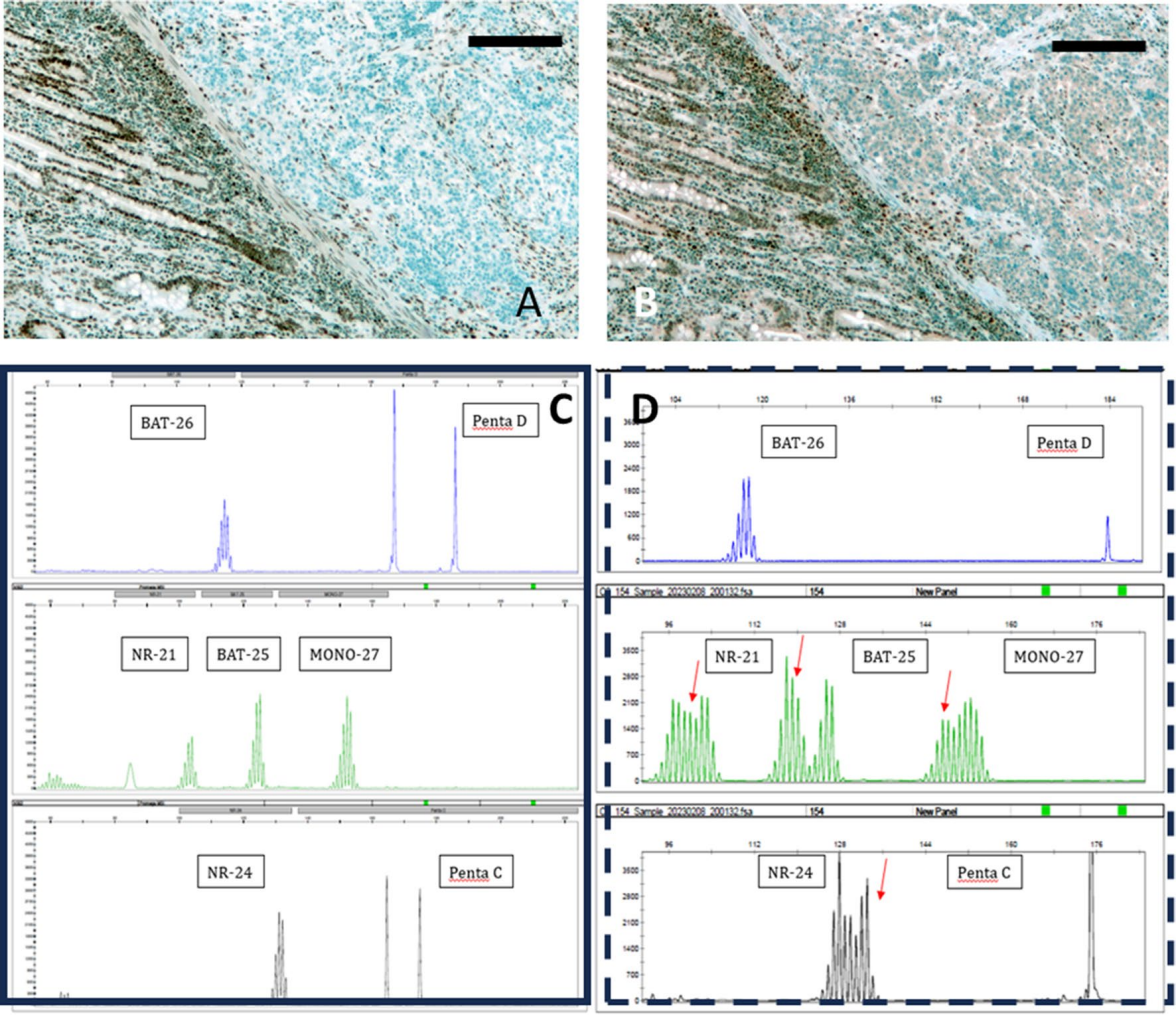
A classical MSI-high colorectal cancer case with MLH1/PMS2 losses. **A**. Loss of MLH1 protein from tumor cells while normal cells remained positive. **B**. Matching area. Loss of PMS2 protein from tumor cells. Bars = 200 µm. C/D. Pentaplex analysis of the case. Tumor proportion: 50%. **C**. Control K562 tumor cell line with all five stable markers. **D**. Tumor sample. Note the aberrant markers BAT-25, Mono27, NR21 and NR24 (red arrows) in the cancer case

**Fig. 2:**
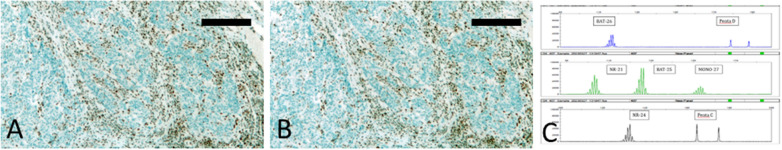
An example of the MSS colorectal cancer case with dual loss of MLH1/PMS2. **A**. Loss of MLH1 protein from tumor cells while normal cells remained positive. **B**. Matching area. Loss of PMS2 protein. Bar = 200 µm. **C**. Pentaplex analysis of the case where none of the 5 markers are aberrant. Tumor proportion: 60%

**Fig.3:**
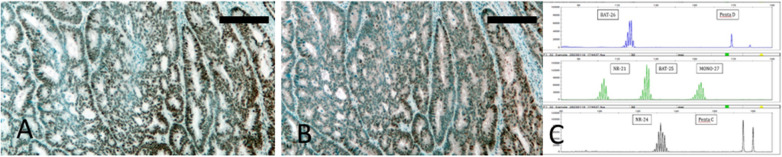
An example of MSS colorectal cancer case with low level heterogenous loss of MLH1/PMS2. **A**. Heterogenous loss of MLH1 protein staining from the nuclei of tumor cells. **B**. Matching area. PMS2 protein positivity in tumor cells. Bar = 200 µm. **C**. Pentaplex analysis of the case where none of the 5 markers are aberrant. Tumor proportion: 70%

We have hypothesized that the phenotype of the dMMR might be a factor influencing the discrepancy, therefore we have subdivided dMMR into classical complete (> 90%) two-protein (MLH1/PMS2 (Figs. 1, 2a, b) or MSH2/MSH6 losses, non-classical single- or multiple complete MMR protein losses and unusual pMMR patterns. (for examples see Additional file 2: Fig. S1a–d and Fig. 3a, b) Next, we have analyzed the correlations of MSI PCR with dMMR phenotypes. This analysis indicated that classical dMMR has a ~ 60% correlation with MSI-H status, the non-classical dMMR has a much lower correlation while the unusual dMMR pattern has a very low (< 10%) correlation with MSI-H in the entire cohort, but also in the colorectal cancer part (Table 5). For a demonstration of MSI/dMMR correlation and discrepancies, please see Figs. 1, 2, 3.

**Table 5:** Comparison of dMMR immunophenotypes and MSI PCR status in the cancer cohort

dMMR	All (n)	Classic	Non-classic	Unusual
all cancers (n)	207	103	55	49
MSS/MSI-L	124	42 (40.8%)	36 (65.5%)	46 (93.9%)
MSI-H	83	61 (59.2%)	19 (34.5%)	3 (6.1%)
colorectal (n)	157	75	43	39
MSS/MSI-L	92	28 (37.3%)	27 (62.8%)	37 (94.9%)
MSI-H	65	47 (62.7%)	16 (37.2%)	2 (5.1%)
non-colorectal (n)	50	28	12	10
MSS/MSI-L	32	14 (50.0%)	9 (75.0%)	9 (90.0%)
MSI-H	18	14 (50.0%)	3 (25.0%)	1 (10.0%)

*dMMR* mismatch repair deficiency, *MSI-H* microsatellite instability-high, *MSI-L* microsatellite instability-low, *MSS* microsatellite stability

Statistical analysis of the dMMR phenotypic variants for correlation with MSI-H indicated that the sensitivity of the PCR in case of the unusual phenotype is extremely low, unlike the specificity (Table 6). On the other hand, there are not much differences in the NPV values of PCR between the other dMMR phenotypes. However, concerning PPV values of PCR, the highest % is in the classic phenotype and in case of the non-classical phenotype the PPV value is very low (Table 6). Analysis of the dMMR/MSI-high correlations by Cohens method indicated a relatively high level (~ 0.7) for the classical phenotype (aspecially in colorectal cancer) and unacceptably lower level (~ 0.5) for non-classical phenotype and the lack of correlation for unusual dMMR IHC patterns. These data are quite similar in the colorectal and non-colorectal cancers (Table 6). When we have calculated the predictive value of MMR IHC for MSI, we have found that classical phenotype has the highest sensitivity, dropping in case of the non-classical phenotype and almost completely lost in case of the unusual phenotype. In parallel to this, the PPV values were very low in case of all these phenotypes while the NPV values remained very high (Additional file 1: Table S4).

**Table 6:** Predictive power of MSI PCR according to the dMMR phenotype

dMMR Phenotype	Sensitivity (%)	Specificity (%)	PPV (%)	NPV (%)	% Agreement	Kappa value
Colorectal						
Classic	62.7	98.5	88.7	93.1	92.6	0.693
Non-classic	37.2	98.5	72.7	93.4	92.3	0.455
Unusual	5.1	98.5	25.0	91.1	89.9	0.056
Non-colorectal						
Classic	50.0	96.4	77.8	88.3	86.9	0.535
Non-classic	25.0	96.4	42.9	92.2	99.3	0.262
Unusual	10.0	96.4	20.0	92.2	89.2	0.082

*kappa* Cohen’s kappa value (95% CI), *MMR* mismatch repair, *NPV* negative predictive power, *PPV* positive predictive power

We have also tested the discrepancy rates in the subcohort where we have excluded unusual dMMR cases and found that the discrepancy rates fell by a third in the entire cohort as well as in the colorectal part, suggesting that one factor influencing this high discrepancy rate is the unusual dMMR phenotype where MSI PCR performance is very poor (Additional file 1: Table S5).

We have also tested if the individual MMR protein types involved in complete losses have any role in the poor performance of the PCR. The two protein losses have been far the most frequent in the dMMR cohort (70%) and the PCR discrepancy was the lowest in case of the MLH1/PMS2 phenotype in case of the colorectal cancers exclusively (Additional file 1: Table S6). The second most frequent protein loss phenotype was MSH2/MSH6 (15%) which was frequently responsible for discrepancy but in colorectal cancers exclusively. The third pMMR IHC phenotype was isolated PMS2 loss (~ 10%) which was also responsible for a significant number of discrepancies in all cancers. Isolated MSH6 loss was rare (~ 5%) and only a small proportion of it was involved in discrepant cases. Any other individual MMR protein losses have a much lower rate (Additional file 1: Table S6).

Next we have questioned if the level of MSI might be a factor affecting discrepancy of dMMR (where we have excluded the unusual cases). We have compared the MSI PCR to dMMR IHC in respect of the extent of the five markers involved (a measure of the level of instability). In colorectal cancer five-marker instability was the most frequent pattern in MSI-H involving 38.1% of the cases, with similar rate of the MSI-L. dMMR cases accumulated in 5-marker instability (more than half of the cases) followed by four marker instability at a much lower level and other marker-patterns were very rare. Interestingly, in non-colorectal cancers, one marker instability was the most frequent (one third of the cases) followed by five-marker instability in PCR. In dMMR cases 5-marker instability was also the most frequent but followed by one-, two and four marker instabilities (Table 7). These data suggest that on the contrary to what would be expected, MSI-L cases represent only a low proportion of dMMR cases (~ 20%) which is much lower than the proportion of MSI-L in the MSI PCR tests.

**Table 7:** Association of level of MSI with dMMR status in the cancer cohort

	MSI-H	MSI-H	MSI-H	MSI-H	MSI-L
Altered markers	**5**	**4**	**3**	**2**	**1**
All cancers					
MSI (147)	56 (38.1%)	21 (14.3%)	5 (3.4%)	9 (6.1%)	56(38.1%)
dMMR (100)	53 (53.0%)	19 (19.0%)	4 (4.0%)	6 (6.0%)	18 (18.0%)
Colorectal					
MSI (114)	46 (40.4%)	18 (15.8%)	3 (2.6%)	4 (3.5%)	43 (37.7%)
dMMR (67)	44 (56.4%)	16 (20.5%)	3 (3.8%)	2 (2.6%)	13 (16.7%)
Non-colorectal					
MSI (33)	10 (30.3%)	3 (9.1%)	2 (6.1%)	5 (15.2%)	13 (39.4%)
dMMR (22)	9 (40.9%)	3 (13.6%)	1 (4.5%)	4 (18.2%)	5 (22.7%)

*dMMR* mismatch repair deficiency, *MSI-H* microsatellite instability-high, *MSI-L* microsatellite instability-low

## Discussion

Predictive diagnostics of immune checkpoint inhibitors includes PDL1 immunohistochemistry and determination of MMR deficiency or MS instability. Since in clinical trials MMR immunohistochemistry or molecular MSI tests were used for patient selection, the registrations consequently included these tests as equal [16, 17]. However, from the viewpoint of immunotherapy efficacy the increased mutational burden and the consequent increase in neoantigens are those factors which influence the efficacy of these therapies. Here we have compared MMR IHC to MSI PCR in a cohort of 700 + cancer cases to see how PCR test results correlate to the MMR IHC. For MMR IHC we have used the international standard 4-protein IHC while for MSI testing we have used also the international standard Pentaplex mononucleotide PCR. Some of the previous large studies found a very high concordance of these tests [19, 20] although nor the IHC neither the PCR testings were homogenous and standardized. In our cancer cohort we have found a relatively high discrepancy rate (~ 19%) which was higher compared to previous studies [22, 23]. Our analysis indicated that the Pentaplex PCR has a high specificity but a very low sensitivity using MMR IHC as standard and the correlation test provided a low value well below the cut-off 0.7. In a previous study using Bethesda-Pentaplex PCR (a less sensitive testing kit) and similar four MMR immunohistochemistry found a similarly high discrepancy rate and low correlation (k = 0.526) [31]. It was expected that the majority of the dMMR discrepancies would fall into the MSI-low category, but that was ~ 8% in colorectal- and ~ 10% in non-colorectal cancers therefore the waste majority were dMMR/MSS discrepancies, suggesting preanalytical factors affecting the PCR testing.

Key factors affecting success of both the MMR IHC and MSI PCR are the technical factors including sample handling, fixation time, nucleic acid quality and quantity [29, 32]. In our case we haven’t used for the comparison such cases where before running the tests technical problems have been identified. Poor performance of the MSI PCR can be due to the low tumor/normal ratio, accordingly, a 20% threshold is suggested by international guidelines [16, 17, 23, 32]. We have repeated our analysis on samples where the T/N ratio was > 20%, but the discrepancy rate was proved to be very similar as in the entire cohort, probably because we have used macrodissection in those cases. Another factor of discrepancy could well be the phenotype of MMR-deficiency: classical, non-classical or unusual [22, 23] Although the positive predictive value of MSI-H for dMMR was the highest (88.7%) in case of classic pattern of colorectal cancers (reaching the recommended correlation k value of 0.7) but was much lower for non-classic pattern (~ 70%) and was extremely low for the unusual variant (25.0%) and these parameters were very similar in non-colorectal cancers as well. It is well known that isolated loss of MSH6 in endometrial cancer is relatively frequent and MSI PCR is not sensitive enough to detect the resulting microsatellite alterations [27, 35]. However, in our series MSH6 isolated loss was rare and only a small proportion of those cases were discrepant. In a recent much smaller study preanalytical factors have been found to be responsible for the high discrepancy rate of dMMR and MSI-H as determined by PCR (k = 0.48) owing to long fixation, low tumor cell content and mucinous tumor histology [32]. In smaller studies relatively high dMMR/MSI-H discrepancy rate was detected (PPV = 0.76%), where the dMMR/MSS non-correlations were found to be similarly low [31–34]. Results of the published literature and our own analysis are questioning the equality of the MMR immunochemistry and the Pentaplex MSI PCR. Alternatives to Pentaplex PCR are the similar Bethesda panel [8, 9], 8 and 10 marker PCR combinations of the Bethesda and the Pentaplex panels [35, 36], completely different 7 and 10 marker sets [35, 36] or various NGS based techniques. [37, 38] These tests have been compared systematically to Pentaplex PCR but only the 7-marker PCR test was compared to MMR IHC [35, 36].

Testing of MMR deficiency was designed to detect Lynch syndrome [8, 9]. We strongly believe that alterations of MMR protein staining or the MS markers by PCR can be a good indication for MMR gene sequencing. However, when these tests are applied for immunotherapy prediction, either positive tests (dMMR or MSI-H) are valid predictors of immunotherapy of patients although the efficacy is linked to increased mutational load due to MSI. Since there are no retrospective or prospective studies on the efficacy of immunotherapy indicated upon dMMR as compared to MSI-H, it cannot be judged the equality/superiority of them. An international cooperation would be needed to clarify these issues.

### Supplementary Information

**Additional file 1: Table S1.** Calculation of the predictive power of MMR IHC for MSI PCR. **Table S2.** Comparison of MMR-IHC and MSI-PCR results in our cancer cohort with T/N ratio >20%. **Table S3.** Pentaplex marker involvement in MSI-status. **Table S4.** Calculation of the predictive power of dMMR phenotypes for MSI PCR. **Table S5.** Comparison of MMR IHC and MSI PCR results in our cancer cohort without unusual dMMR cases. **Table S6.** Distribution of individual MMR protein loss types (>90%) among MSS/MSI-low discrepant cases.

**Additional file 2: Figure S1.** Demonstration of various unusual dMMR phenotypes in MSI PCR discrepant cases.

## Data Availability

Raw data of this study are available at the corresponding author upon request.

## Abbreviations

CRC: Colorectal cancer
CUP: Cancer of unknown primary
FFPE: Formalin fixed paraffin embedded
ICI: Immunecheckpoint inhibitor
IHC: Immunohistochemistry
dMMR: Mismatchprotein deficiency
pMMR: Maintained mismatch protein expression
MSI: Microsatellite instability
MSS: Microsatellite stability
NPV: Negative predictive value
PPV: Positive predictive value
TMB: Tumor mutation burden
