# Supplementary material for: Comparison of standard mismatch repair deficiency and microsatellite instability tests in a large cancer series
Source: J Transl Med. 2024 Feb 13;22:150. doi: 10.1186/s12967-024-04960-y (PMC10863158; doi:10.1186/s12967-024-04960-y)
Supplement: Supplementary file 1 — Additional file 1: Table S1. Calculation of the predictive power of MMR IHC for MSI PCR. Table S2. Comparison of MMR-IHC and MSI-PCR results in our cancer cohort with T/N ratio >20%. Table S3. Pentaplex marker involvement in MSI-status. Table S4. Calculation of the predictive power of dMMR phenotypes for MSI PCR. Table S5. Comparison of MMR IHC and MSI PCR results in our cancer cohort without unusual dMMR cases. Table S6. Distribution of individual MMR protein loss types (>90%) among MSS/MSI-low discrepant cases. [file 12967_2024_4960_MOESM1_ESM.docx]

*Supplementary Table 1. Calculation of the predictive power of MMR IHC for MSI PCR.*

| cancer type | sensitivity (%) | specificity (%) | PPV (%) | NPV (%) |
| --- | --- | --- | --- | --- |
| colorectal | 91.6 | 80.5 | 41.4 | 98.5 |
| non-colorectal | 80.0 | 75.7 | 32.0 | 96.4 |

MMR IHC= mismatch protein immunohistochemistry, MSI=microsatellite instability, NPV= negative predictive power, PPV= positive predictive power

*Supplementary Table 2. Comparison of MMR IHC and MSI PCR results in our cancer cohort with T/N ratio >20%*

|  | n | MSS  n | MSI-L  n | MSS/MSI-L  n | MSI-H  n | discrepancy n(%) |
| --- | --- | --- | --- | --- | --- | --- |
|  |  |  |  |  |  |  |
| all cancers | 506 | 390 | 44 | 434 | 72 | 96 (18.9%) |
| pMMR | 352 | 312 | 33 | 345 | 7 | 7 |
| dMMR | 154 | 78 | 11 | 89 | 65 | 89 |
|  |  |  |  |  |  |  |
| colorectal | 397 | 308 | 34 | 342 | 55 | 69 (17.4%) |
| pMMR | 279 | 250 | 26 | 276 | 3 | 3 |
| dMMR | 118 | 58 | 8 | 66 | 52 | 66 |
|  |  |  |  |  |  |  |
| non-colorectal | 109 | 82 | 10 | 92 | 17 | 27 (24.8%) |
| pMMR | 73 | 62 | 7 | 69 | 4 | 4 |
| dMMR | 36 | 20 | 3 | 23 | 13 | 23 |

MMRD= mismatch repair deficiency, MMRP= microsatellite proficiency, MSI-H= microsatellite instability-high, MSI-L= microsatellite instability-low, MSS= microsatellite stability, MSS= microsatellite stable, N= normal, T=tumor

*Supplementary Table 3. Pentaplex marker involvement in MSI-status*

| markers | BAT25 (n/%) | BAT26  (n/%) | mono27  (n/%) | NR21  (n/%) | NR24  (n/%) |
| --- | --- | --- | --- | --- | --- |
| all cancers (147) | 114 | 79 | 83 | 94 | 83 |
| MSI-H (91) | 85 (93.4) | 76 (83.5) | 80 (87.9) | 79 (86.8) | 77 (84.6) |
| MSI-L (56) | 29 (51.8) | 3 (5.4) | 3 (5.4) | 15 (26.8) | 6 (10.7) |
| colorectal (114) | 91 | 63 | 65 | 76 | 67 |
| MSI-H (71) | 67 (94.4) | 61 (85.9) | 64 (90.1) | 64 (90.1) | 63 (88.7) |
| MSI-L (43) | 24 (55.8) | 2 (4.7) | 1 (2.3) | 12 (27.9) | 4 (9.3) |
| non-colorectal (33) | 23 | 16 | 18 | 18 | 16 |
| MSI-H (20) | 18 (90.0) | 15 (75.0) | 16 (80.0) | 15 (75.0) | 14 (70.0) |
| MSI-L (13) | 5 (38.5) | 1 (7.7) | 2 (15.4) | 3 (23.1) | 2 (15.4) |

MSI-H= microsatellite instability-high (2-5 markers), MSI-L= microsatellite instability-low (1 marker)

*Supplementary Table 4. Calculation of the predictive power of dMMR phenotypes for MSI PCR.*

|  | sensitivity (%) | specificity (%) | PPV (%) | NPV(%) |
| --- | --- | --- | --- | --- |
| CRC |  |  |  |  |
| CL | 88.7 | 93.1 | 62.7 | 98.5 |
| NCL | 72.7 | 93.4 | 37.2 | 98.5 |
| UN | 25.0 | 91.1 | 5.1 | 98.5 |
| non-CRC |  |  |  |  |
| CL | 77.8 | 88.3 | 50.0 | 96.4 |
| NCL | 42.9 | 92.2 | 25.0 | 96.4 |
| UN | 20.0 | 92.2 | 10.0 | 96.4 |

CL= classic phenotype, CRC= colorectal cancer, dMMR= mismatch repair protein deficiency, MSI= microsatellite instability, NCL= non-classic phenotype, NPV= negative predictive power, PPV= positive predictive power, UN= unusual phenotype

*Supplementary Table 5. Comparison of MMR IHC and MSI PCR results in our cancer cohort without unusual dMMR cases.*

|  | n | Discrepancy  n(%) | MSS  n(%) | MSI-L  n(%) | MSI-H  n(%) |
| --- | --- | --- | --- | --- | --- |
| all cancers | 480 | 65 (13.5%) |  |  |  |
| pMMR | 355 | 5 (1.4%) | 320 (90.1%) | 30 (84.5) | 5 (1.4%) |
| dMMR | 125 | 60 (48.0%) | 55 (44.0%) | 5 (4.0%) | 65 (52.0%) |
| colorectal | 374 | 47 ((12.6%) |  |  |  |
| pMMR | 280 | 3 (1.1%) | 254 (90.7%) | 23 (8.2%) | 3 (1.1%) |
| dMMR | 95 | 44 (46.3%) | 40 (42.1%) | 4 (4.2%) | 51 (53.7%) |
| non-colorectal | 80 | 12 (15.0%) |  |  |  |
| pMMR | 58 | 1 (1.7%) | 52 (89.7%) | 5 (8.6%) | 1 1.7%) |
| dMMR | 22 | 11 (50.0%) | 10 (45.5%) | 1 (4.5%) | 11 (50.0%) |

dMMR= mismatch repair deficiency, pMMR= microsatellite proficiency, MSI-H= microsatellite instability-high, MSI-L= microsatellite instability-low, MSS= microsatellite stability, MSS= microsatellite stable

*Supplementary Table 6. Distribution of individual MMR protein loss types (>90%) among MSS/MSI-low-discrepant cases.*

| protein loss | PMS2 | MLH1 | MSH2 | MSH6 | 2P | MLH1  PMS2 | MSH2  MSH6 | 3P | 4P |
| --- | --- | --- | --- | --- | --- | --- | --- | --- | --- |
| CRC (118) | 12 | 6 | 2 | 7 | 82 | 58 | 17 | 5 | 4 |
| MSS/MSIL | 9 | 3 | 1 | 2 | 32 | 18 | 10 | 4 | 4 |
| MSI-H | 3 | 3 | 1 | 5 | 50 | 40 | 7 | 1 |  |
| non-CRC (42) | 5 | 1 | 2 | 2 | 30 | 21 | 7 | 2 |  |
| MSS/MSIL | 4 | 1 | 2 | 1 | 15 | 12 | 2 | 1 |  |
| MSI-H | 1 |  |  | 1 | 15 | 9 | 5 | 1 |  |

CRC= colorectal cancer, MSIL= microsatellite instability low, MSI-H= microsatellite instability high, MSS= microsatellite stability, P= MMR protein
