# Supplementary figures and images for: Comparison of standard mismatch repair deficiency and microsatellite instability tests in a large cancer series
Source: J Transl Med. 2024 Feb 13;22:150. doi: 10.1186/s12967-024-04960-y (PMC10863158; doi:10.1186/s12967-024-04960-y)

## Slide 1
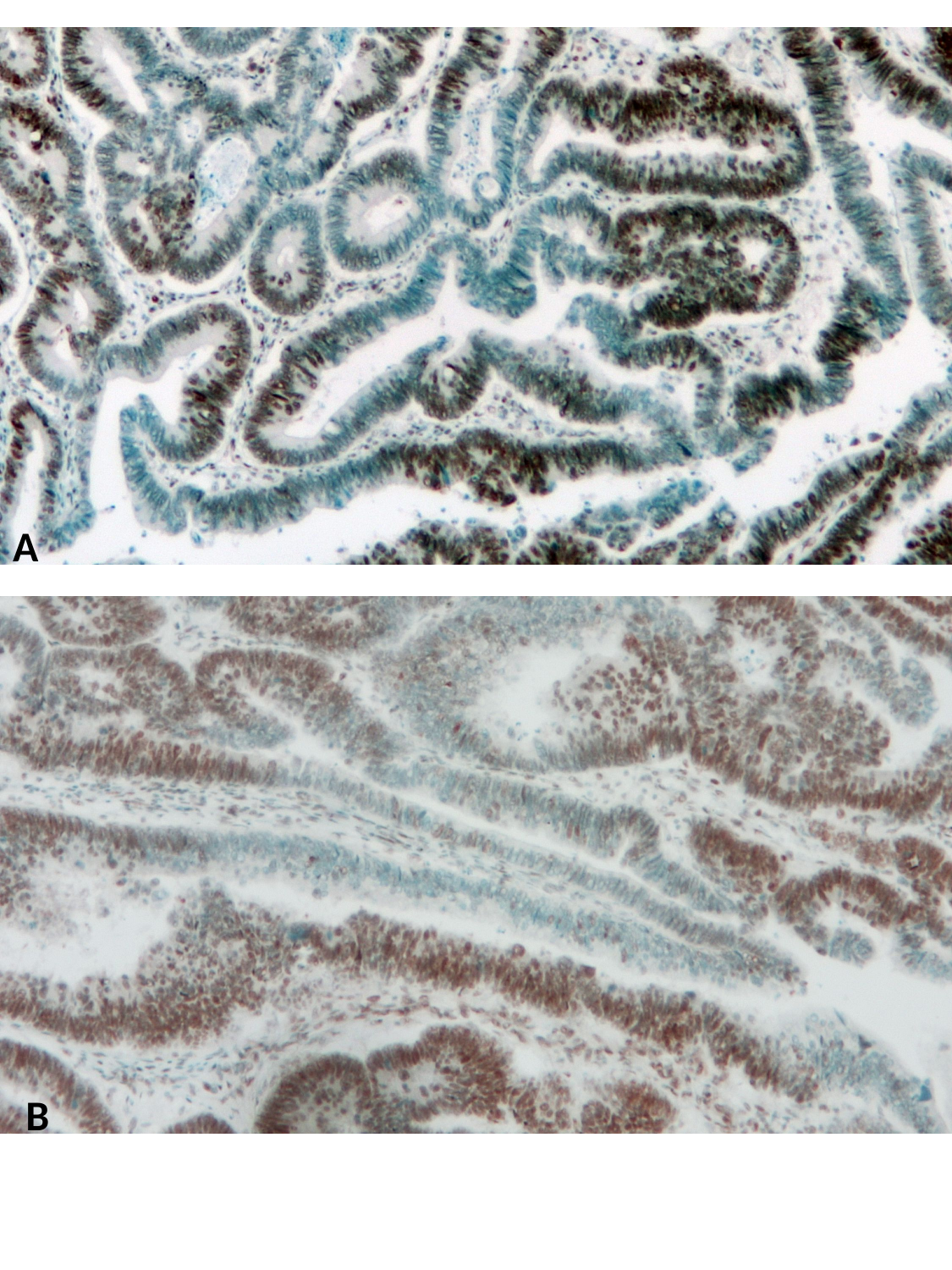

A
B

## Slide 2
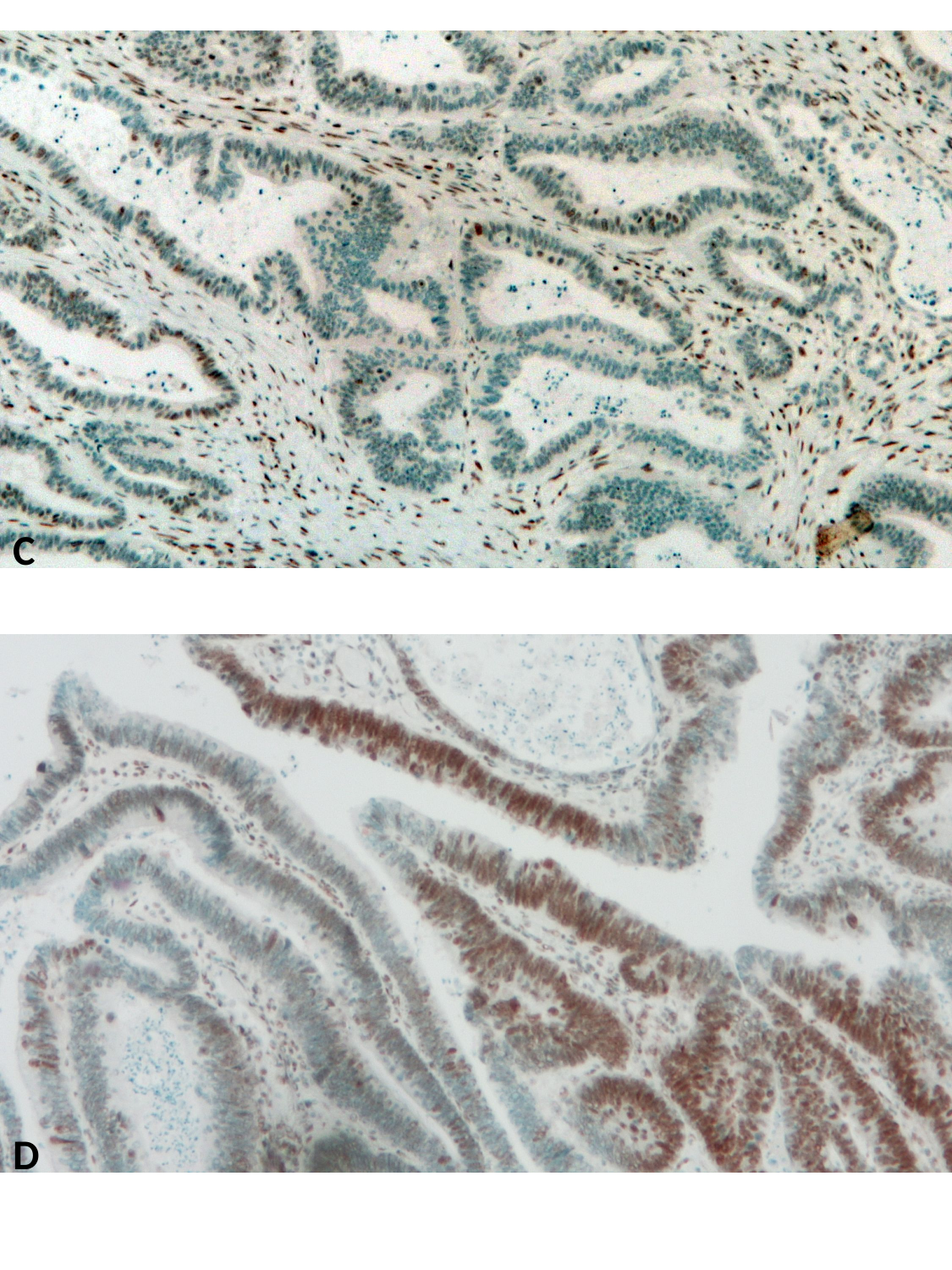

C
D

Supplement: Supplementary file 2 — Additional file 2: Figure S1. Demonstration of various unusual dMMR phenotypes in MSI PCR discrepant cases. [file 12967_2024_4960_MOESM2_ESM.pptx]
